# Perceptions of Endocrine Clinicians Regarding Climate Change and Health

**DOI:** 10.3390/ijerph22020139

**Published:** 2025-01-21

**Authors:** Samantha Steinmetz-Wood, Amanda G. Kennedy, Juvena R. Hitt, Kaitlyn Barrett, Matthew P. Gilbert

**Affiliations:** 1 Division of Endocrinology, Diabetes & Osteoporosis, The University of Vermont Larner College of Medicine, Burlington, VT 05401, USA; kaitlyn.barrett@uvmhealth.org (K.B.); matthew.gilbert@uvmhealth.org (M.P.G.); 2 Department of Medicine Quality Program, The University of Vermont Larner College of Medicine, Burlington, VT 05401, USA; amanda.kennedy@uvmhealth.org (A.G.K.);

**Keywords:** climate change, endocrine clinicians, health impacts, survey

## Abstract

The effects of climate change on the endocrine system are increasingly recognized. We aimed to evaluate endocrine clinicians’ perspectives on climate change awareness and knowledge, motivation for action, and the need for climate health curricula. We designed an online questionnaire with endocrine-specific questions about climate change, which was shared through social media and email. Study data were collected between 9/2022 and 11/2022. Analyses were primarily descriptive. There were 164 responses; 98% were physicians, with a median age of 41 years. The majority (95%) reported that climate change is happening; 52% reported that they are very worried. Knowledge about climate change and health was variable (6.7% very, 40% moderately, 35% modestly, 17.7% not at all), with variable concerns regarding patient effects. The top endocrine climate–health concerns were reduced exercise, malnutrition, and weather-related disruptions. Most respondents agreed that climate change and health topics should be integrated into medical education (72.8% strongly agree or agree). The three resources perceived as most helpful were continuing medical education, patient resources, and policy statements. Endocrine clinicians are aware of and worried about climate change, with varying levels of knowledge and concern about climate change and health effects. We also exposed an untapped interest in developing endocrine-specific climate and health curricula.

## 1. Introduction

The threat of climate change and environmental pollutants to health is increasingly recognized in the medical community [1,2]. In September 2021, more than 200 health journals, in a simultaneous publication, called for emergency action on the climate crisis, stating “as health professionals, we must do all we can to aid the transition to a sustainable, fairer, resilient, and healthier world”, and calling climate change “the greatest threat to global public health” [1]. In June 2022, the American Medical Association announced a policy declaring climate change a public health crisis [2]. There is also growing support for education on climate change, including a global policy report that recommends incorporating climate change education into medical school curricula to help physicians understand the climate emergency and its health impacts [3]. Preventing the effects of climate change on health is also a major motivator for patients. A 2019 ecoAmerica survey found that 66% of Americans believe that if the United States took steps to prevent climate change, it would improve their health and 76% chose “protecting personal and public health” as their top motivation for supporting climate solutions [4]. The survey also indicated that 64% of Americans trust health professionals for information on climate change; however, only 19% of Americans report recently hearing about climate change from health professionals [4].

There is a growing evidence base describing the environmental insults and harms of climate change and human-made pollutants on the endocrine system [5,6,7,8,9,10,11,12,13]. Key environmental threats to the endocrine system include endocrine-disrupting chemicals, the effects of air pollution, such as the associations of particulate matter of 2.5 microns or less in diameter (PM_2.5_) with diabetes incidence and prevalence, cortisol and catecholamine levels, maternal thyroid function tests, as well as the effects of air pollution on vitamin D deficiency [5,6,7,8,9,10,11,12,13].

Individuals with diabetes, a main focus of endocrine care, are particularly vulnerable to the effects of climate change [10,14]. One study found an increase in diabetes incidence with higher temperatures [15]. A Spanish study found that elevated ambient temperatures were associated with an increased prevalence of dysglycemia and insulin resistance in a large cohort of adults, which could only be partially explained by changes in physical activity [16]. Hot weather and heat waves have been associated with increased admissions and emergency room visits among individuals with diabetes [17,18]. Moreover, a Brazilian study estimated that every 5 °C increase in daily mean temperature was associated with a 6% increase in hospitalization due to diabetes [17]. Diabetic patients are also more prone to dehydration and heatstroke [10,14,19]. Studies have also shown associations between air pollution and increased insulin resistance, as well as an increased incidence of diabetes [10,20,21]. More acutely, after exposure to fine particulate matter (PM_2.5_) from a wildfire event, individuals with diabetes were found to have an increased risk of respiratory and cardiovascular physician visits in the period after a wildfire [10,22].

There is an underlying interplay between sustaining the modern human diet, human health, and associated environmental impacts [23]. Agriculture is responsible for approximately one-third of greenhouse gas emissions, mainly produced by methane from cattle and nitrous oxides from fertilizer, and food systems are a leading cause of land conversion, deforestation, and loss of biodiversity [5,24]. The overconsumption of unhealthy processed foods and animal-based food consumption is linked to increased rates of obesity, diabetes, cancer, and cardiovascular disease [25]. In addition, the way that food is raised, prepared, processed, and packaged influences exposure to endocrine-disrupting chemicals [26]. Recently, the endocrine community has also started to publicly recognize the threat of climate change to endocrine health. In 2022, the Endocrine Society announced the goal “to increase awareness of the impact of climate change on endocrine health” [27], and the 2023 Endocrine Society Conference featured plenaries focusing on the health impacts of climate change.

Endocrine professionals routinely strive to provide safe and effective care to their patients while providing preventative care aimed at ensuring a healthy future for their patients. Endocrine clinicians have a unique perspective and opportunity to understand the health effects of environmental pollution and climate change, to assume leadership in a preventative and educational role in climate preparedness, and to treat health outcomes related to climate change and environmental pollution. Despite the existing impacts of climate change and environmental threats on human endocrine health, there is little information on the viewpoints of practicing endocrine clinicians regarding this topic. To the best of our knowledge, there is no existing survey assessing the perceptions of endocrine clinicians on climate change and health. The purpose of this study was to evaluate endocrine clinicians’ perceptions of climate change awareness and knowledge, as well as their motivation and barriers to incorporating climate change concepts into practice, and to demonstrate the need for climate change curricula in endocrine training.

## 2. Methods

This study included a 5 min questionnaire for endocrinology clinicians. The survey questions were developed from a review of previously published surveys that assess physicians’ experiences with climate change [28,29,30], selecting questions that focus on the domains of climate change awareness and knowledge, as well as motivation for action. Questions addressing demographics, endocrine-specific topics, and perceptions of climate health curricula in endocrine training were added. The survey contained 18 questions (Table S1), primarily consisting of Likert-scale questions, along with some multiple-choice questions and 1 free-text response question at the end of the survey, where respondents were given the opportunity to provide an anecdote about their experiences or comment freely on the subject. The final questionnaire was pilot tested with the endocrine clinicians in our clinic for completion time, readability, and overall flow of questions but was not otherwise validated. Data were collected and managed using REDCap electronic data capture tools hosted at the University of Vermont [31]. This study was approved as exempt research by the University of Vermont Committees on Human Research (STUDY00002229).

Eligible participants included self-identified endocrinology clinicians (e.g., MD, DO, diabetes educators, nurse practitioners, and physician assistants). Non-medical participants and participants who do not work in an endocrine practice were excluded. A link to the Redcap survey was sent to members of the endocrine community through multiple methods, including social media (Facebook Endocrinologist group, WhatsApp Endocrine fellow group, Twitter, DocMatter Endocrine Society page), and an email was sent to all endocrine fellowship program directors within the United States.

We shared the link via the listed methods 2 times in hopes of maximizing recruitment. The link opened an information sheet describing the purpose of this study and its procedures. Participants were told that they would be asked a series of questions about their endocrine practice and their perceptions of climate change. Participants were also told that this was a one-time, de-identified questionnaire. The information sheet concluded with a yes or no question about whether the participant would like to proceed with the study. Those who indicated they would like to proceed were directed to the actual questionnaire. We closed the survey when no new surveys arrived. Study data were collected between September 2022 and November 2022.

Data were analyzed using descriptive and univariate statistics. Continuous data were analyzed using Wilcoxon rank sum tests, and Fisher’s exact test or chi-square analyses were used for categorical data. Analyses were conducted using STATA 16.1 (Stata Corporation, College Station, TX, USA), with *p* < 0.05 required for statistical significance.

## 3. Results

A total of 164 self-identified endocrinology clinicians completed the online questionnaire (Table 1). A total of 64% of participants identified as female, and 98% of respondents were physicians; among these, 31% were program directors, and 29% were endocrine fellows. The median age was 41 years (mean 44 years), and 91% reported being employed in the United States. The majority indicated both outpatient and inpatient settings as their primary work environment (58%).

The majority of respondents (95%) reported that climate change is happening, and 52% were very worried about climate change. Female clinicians were significantly more worried about climate change than male clinicians (*p* = 0.02). Responses were variable regarding knowledge about climate change and health (7% very, 40% moderately, 35% modestly, and 18% not at all) and concerns about the effects of climate change on patient health (13% a great deal, 36% a moderate amount, 26% only a little, 8% not at all, 17% don’t know). The top three endocrine climate–health concerns identified were reduced exercise due to motorized transport, malnutrition resulting from food prices, and disruptions to healthcare services during weather events (Table 2). The majority of respondents reported motivation to take action in their personal or professional lives regarding climate change (69% strongly agree or agree). Free-text comments reflected a variety of opinions, with selected examples presented in Table 3.

There were significant differences in responses based on age. Compared to older clinicians, younger clinicians (aged less than 44 years) were significantly more concerned about global climate change affecting patients in terms of anxiety, depression, or other mental health conditions (*p* = 0.003); increased poverty due to economic hardship and resulting health problems (*p* = 0.001); disease incidence and severity related to exposure to particulate matter from air pollution (*p* = 0.02); disruptions to health care services for people with chronic conditions during extreme weather events (*p* = 0.009); the effects of increased meat consumption on patient health (*p* = 0.04); and the environmental effects of medical waste (*p* = 0.02). Younger clinicians were also significantly more motivated to take action in their personal or professional lives (*p* = 0.006).

Responses were divided on whether clinicians have a responsibility to bring the health effects of climate change to their patients’ attention (12% strongly agree, 37% agree, 40% neutral, 6% disagree, 5% strongly disagree). The majority have rarely (38%) or never (45%) discussed climate change with their patients. The three most highly ranked barriers to addressing climate change-related health topics with patients included lack of time (66%), lack of knowledge on how to approach the issue with patients (48%), and the clinician’s lack of knowledge on the subject (45%). The three resources perceived as most helpful were continuing medical education (CME), patient education materials, and policy statements.

The majority of respondents agreed that teaching about climate change and health impacts should be integrated into medical education (73% strongly agree or agree), and 83% of the endocrine program directors and fellows indicated that their program does not cover this topic. Both program directors and fellows agreed, without a significant difference (*p* = 0.13), that teaching about climate change and its association with health impacts should be integrated into medical education.

## 4. Discussion

We found that the majority of endocrine clinicians who responded to the survey were aware of and worried about climate change, with variable degrees of knowledge about the topic. The top three endocrine climate–health concerns were reduced exercise due to motorized transport, malnutrition linked to food prices, and disruptions to healthcare services during weather events. Most respondents agreed that teaching about climate change and its health effects should be integrated into medical education, with consensus among program directors and fellows; however, according to our survey results, the majority of endocrine fellowship programs do not teach about climate change and its associations with health.

We noted that the effects of increased meat consumption on the health of patients, as well as the climate effects from farming animals related to high rates of meat consumption, did not rank among the top three endocrine climate–health concerns. We had expected this to be a top concern given the important relationships between diet and endocrine health conditions, such as diabetes, as well as the effects of agriculture on planetary health. Furthermore, it has been recognized that a shift to healthy and sustainable plant-forward diets would have significant benefits in both reducing greenhouse gas emissions and improving health outcomes [32,33,34]. Endocrine clinicians already frequently counsel patients on healthier diets and physical activity. There is also an opportunity to engage patients in these activities from a more sustainable perspective. On an individual level, adopting a plant-based diet can save 0.8 tonnes of CO_2_-equivalent emissions per year, which is considered a high-impact action that substantially reduces personal emissions [35]. It has been found that individuals’ willingness to eat less meat increases with its perceived effectiveness [36], which hints at the importance of increasing education and awareness on these topics.

In addition, encouraging exercise or the use of public transport over individual motorized transport can have substantial effects on both the environment and individual health. Increased public transport usage is associated with a decreased prevalence of obesity [37]. Commuting by bicycle or walking can decrease greenhouse gas emissions, and in turn, bicycle commuters experience an estimated 50% reduction in all-cause mortality and cardiovascular disease [5,38]. Reduced exercise due to motorized transport was a top concern among endocrine clinicians who responded to the survey.

We noted some significant differences in responses according to age group, with younger clinicians reporting both more concerns and more motivation to take action regarding climate change. This may be aligned with a generation gap in climate change beliefs [39]. However, this contrasts with some studies that report positive relationships between age and pro-environmental behaviors. We do not have sufficient detail in the questionnaire to explain why the differences in responses by age exist. A qualitative or mixed methods study would likely be required to explore these findings. These results should be explored in future research, as interventions to address climate change may vary by age group.

The finding that 5% of surveyed endocrinology clinicians do not accept that climate change is happening is concerning. Future research should further explore the perceptions and characteristics of clinicians who do not believe climate change is occurring.

We would also like to highlight that the gap in healthcare education regarding the effects of climate change on health also affects the endocrine community. The low percentage of respondents (7%) who feel “very knowledgeable” about the connection between climate change and health is concerning. This gap in understanding can hinder effective advocacy and action. Our survey revealed a self-reported knowledge gap, with 17.7% of respondents indicating that they feel not at all knowledgeable about climate change and its health impacts. We also found that the majority of respondents, many of whom are program directors or fellows, agreed that teaching about climate change and health impacts should be integrated into medical education, and 83% of the program directors and fellows indicated that their programs do not cover this topic. As concisely stated in a Lancet comment on healthcare education, “It is time for a global planetary health education revolution to equip the health sector to treat the Code Red Emergency we face” [40]. Without intentionally creating educational opportunities on planetary health topics, we are missing opportunities to equip endocrine clinicians with the knowledge base and tools needed to address this threat as we continue to encounter the effects of climate change. Climate change and health topics should be incorporated into endocrine fellowship curricula and CME activities.

Responses were divided on whether clinicians have a responsibility to bring the health effects of climate change to the attention of their patients. Similar to a multinational survey on the views of health professionals on climate change and health [29], a lack of time was the most common barrier identified. Other important barriers included a lack of knowledge on how to approach the issue with patients and the clinician’s own lack of knowledge on the subject. This survey serves as a starting point for understanding the perspective of endocrine clinicians on climate change and health and for identifying gaps in knowledge, awareness, and self-efficacy. There is evidence suggesting that informing people about the health effects of climate change, as well as solutions to address them, can increase support for actions to reduce emissions [29,41,42].

There were limitations. This was a non-sponsored survey, which limited the sample size, as there were no incentives for completion. Our data allowed us to identify some factors affecting concerns about climate change and health among endocrine clinicians, such as age and gender. A larger study with more data may allow for the exploration of other interesting associations between responses, such as the effects of geographical location or education level. It is possible that the clinicians who responded were more concerned about climate change than those who did not complete the survey. There may also be selection bias, given that not all endocrine clinicians use social media, and younger individuals may be more likely to use it. The questionnaire included questions from prior published surveys primarily focused on the themes of awareness and knowledge, along with additional questions of interest. We acknowledge that our final survey did not undergo the full validation process, other than pilot testing to ensure the clarity and functionality of the survey interface. The endocrine topics incorporated into the survey are documented endocrine-related health concerns found in the literature. Undoubtedly, there are more endocrine health concerns related to climate change or environmental health that have not been included in the survey. We also did not specifically mention important issues surrounding climate change that exacerbate health and social inequities, such as the unequal exposure of racial or socioeconomic groups to air pollution or power imbalances in the food system. This survey does not provide evidence that the specific health concerns described are directly climate- or endocrine-related. Despite its limitations, this survey brings together documented climate and endocrine-related health effects and the opinions of current practicing clinicians and adds to the body of evidence regarding climate change perceptions.

Ultimately, clinicians should aim to deliver sustainable services that augment wellbeing and reduce health inequalities. Endocrine clinicians, as health care workers, are trusted voices within the community and have the opportunity to encourage healthy collective behaviors for sustainable living and even have the potential to be strong advocates for sustainable healthcare systems and climate action. Examples of smaller actions that can still have a meaningful impact include encouraging patients to take nature walks, encouraging sustainable eating, prescribing re-usable insulin pens, reducing unnecessary bloodwork, integrating telemedicine into practice, discussing climate issues with colleagues, and developing emergency action plans for diabetes patients during heat waves or natural disasters.

## 5. Conclusions

The majority of the endocrine clinicians surveyed were aware of and worried about climate change, with varying levels of knowledge and concern about climate change and its health effects. Most respondents agreed that teaching about climate change and health effects should be integrated into medical education, with similar responses among program directors and fellows. In addition, most reported being motivated to take action in some way for climate change. The results also reflect an untapped interest in developing a curriculum focused on climate change and endocrine health within fellowship programs and CME.

## Figures and Tables

**Table 1:** Survey Participant Demographics (N = 164).

Age Distribution (Years)	N	(%)
≤35	49	(30)
36–45	46	(28)
46–55	32	(20)
56–65	19	(12)
≥66	18	(11)
**Gender Identity**		
Female	105	(64)
Male	57	(35)
Missing	2	(1)
**Location**		
United States	149	(91)
Northeast	35	(23)
South	38	(26)
Midwest	17	(11)
West	26	(17)
Did not specify	33	(22)
Other Country/Missing	15	(9)
**Role**		
Physician	161	(98)
Program Director	51	(31)
Fellow	47	(29)
Other	3	(2)
**Work Setting**		
Outpatient	65	(40)
Inpatient	2	(1)
Both	95	(58)
Other/Missing	2	(1)

**Table 2:** Perceptions of global climate change and its impact on patients *.

	A Moderate Amount or a Great Deal		Not at All or Only a Little	
	N	(%)	N	(%)
Reduced exercise due to excessive motorized transport	139	(85)	19	(12)
Hunger and malnutrition due to rising food prices	135	(83)	21	(13)
Disruptions to health care services for people with chronic conditions during extreme weather events	123	(75)	35	(21)
Increased poverty due to economic hardship, and resulting health problems	120	(74)	32	(20)
Disease incidence and severity related to exposure to particulate matter from air pollution	116	(71)	35	(21)
Effects of increased meat consumption on health of patients	106	(65)	38	(23)
Climate effects from farming animals related to high rates of meat consumption	104	(63)	41	(25)
Exposure to endocrine-disrupting chemicals	104	(63)	40	(24)
Anxiety, depression, or other mental health conditions	102	(62)	41	(25)
Environmental effects of medical waste	97	(60)	42	(26)
Extreme heat effects on reproductive and maternal health	79	(49)	48	(30)
Vitamin D deficiency related to air pollution	71	(44)	65	(40)
Effects of extreme temperature and climate on thyroid function	45	(27)	83	(50)

* Remaining respondents in each category answered “don’t know”or did not respond.

**Table 3:** Selected narrative comments from survey respondents *.

While the effects of natural disasters are easy to communicate to patients/faculty, more insidious climate changes are more difficult to convey and use of education/examples would benefit both providers and patients.
Summers are increasingly hotter, making less likely for Pts [patients] to take a walk or exercise.
Should be careful that focusing on the effort of single providers don’t [sic] distract you from the actions that have proven to benefit climate, which tend to be policy/governmental changes.
Personally I believe climate change is a vital topic that needs to be dealt with urgently, however, I do not believe the exam room is where we should discuss this topic. Most patients who see an endocrinologist have complex issues, require complex regimens, etc. When they walk through the door they are already anxious and have difficulty focusing on what we are telling them.
Not sure that individuals can do much about climate change…
Lack of water is going to be a major issue in my lifetime. We need to take action now to prevent these changes, but it’s hard because it’s the “upstream” folks that need to make the biggest changes and they don’t see a problem yet.
I lived in [the South] where we’ve had electric grid issues as well as outages due to hurricanes in the past 2 years. It has been traumatizing to see how our poverty-stricken area is affected by these outages. Accessibility to care, both for doctors and patients, is threatened when this happens. I worry about increased flooding and storms.
I act frequently on climate-related issues in my personal life but haven’t brought this into work.
Given the numerous impacts of climate on health it is difficult to find which information is accurate and appropriate. Medical education should start with climate impacts that have been proven.

* Free-text comments were provided in response to the following question: “Is there anything else you would like us to know?”.

## Data Availability

Some or all datasets generated during and/or analyzed during the current study are not publicly available but are available from the corresponding author upon reasonable request.

## Supplementary Materials

The following supporting information can be downloaded at https://www.mdpi.com/article/10.3390/ijerph22020139/s1: S1. Climate change survey.
