# Supplementary material for: Perceptions of Endocrine Clinicians Regarding Climate Change and Health
Source: Int J Environ Res Public Health. 2025 Jan 21;22(2):139. doi: 10.3390/ijerph22020139 (PMC11855314; doi:10.3390/ijerph22020139)
Supplement: Supplementary file 1 [file ijerph-22-00139-s001.zip › ijerph-3392938-supplementary.pdf]

Supplementary Materials

S1. Climate Change Survey

**Question**	
1.	What role best describes you? (physician, diabetes educator, nurse practitioner, physician assistant, other clinician)
1a.	Are you a program director, association program director, fellow, neither?
2.	Which best describes your practice or type of endocrine work? (general endocrinology, pediatric endocrinology, sub-specialist)
3.	What is, or if retired was, your primary work setting? (outpatient, inpatient, both, other)
4.	What is your gender identity? (female, male, prefer to self-describe, prefer not to respond)
5.	What is your age? (years)
6.	In which country do you (or did you) work? (United States, Canada, other)
7.	Do you think that climate change is happening? (Yes, No)
	If yes: How sure are you that climate change is happening? (Extremely sure, Very sure, Somewhat sure, Not at all sure)
	If no: How sure are you that climate change is not happening? (Extremely sure, Very sure, Somewhat sure, Not at all sure)
8.	How worried are you about climate change? (Very worried, Somewhat worried, Not too worried, Not at all worried)
9.	How knowledgeable do you feel about the association between climate change and health impacts? (Very knowledgeable, Moderately knowledgeable, Modestly knowledgeable, Not at all knowledgeable)
10.	How much, if at all, do you think climate change is affecting the health of your patients? (A great deal, A moderate amount, Only a little, Not at all, Don't know, I don't currently see patients)
11.	How do you believe that global climate change has affected or will affect your patients from an endocrine perspective? (Not at all, Only a little, A moderate amount, A great deal, Don't know)
	• Anxiety, depression or other mental health conditions
	• Increased poverty due to economic hardship and resulting health problems
	• Disease incidence and severity related to exposure to particulate matter from air pollution
	• Disruptions to health care services for people with chronic conditions during extreme weather events
	• Exposure to endocrine disrupting chemicals
	• Vitamin D deficiency related to air pollution
	• Effects of extreme temperature and climate on thyroid function
	• Effects of increased meat consumption on health of patients
	• Climate effects from farming animals related to high rates of meat consumption
	• Hunger and malnutrition due to rising food prices
	• Reduced exercise from excessive motorized transport
	• Environmental effects of medical waste
	• Extreme heat effects on reproductive and maternal health
12.	Which of the following, if any, are barriers that prevent you from addressing climate change-related or health issues with patients? (check all that apply)
	• Climate change is not occurring
	• My patients would not be interested or knowledgeable enough about climate impacts to discuss this issue

	• Lack of time
	• Lack of my knowledge
	• Lack of knowledge regarding how to approach the issue with my patients
	• It won't make a difference if I do
	• Topic is too controversial
	• Lack of support from my peers
	• Other barriers
13.	Teaching about climate change and its association with health impacts should be integrated into medical education. (Strongly agree, Agree, Neutral Disagree, Strongly disagree)
13a.	Our fellowship program teaches about climate change and its association with health impacts. (Yes, No, Unsure)
14.	I feel motivated to take action in my personal and/or professional life to contribute to efforts for climate change. (Strongly agree, Agree, Neutral Disagree, Strongly disagree)
15.	Clinicians have a responsibility to bring the health effects of climate change to the attention of their patients. (Strongly agree, Agree, Neutral Disagree, Strongly disagree)
16.	How often have you discussed climate change with your patients? (Never, Rarely, Sometimes, Often, Always)
17.	Which of the following resources, if any, would be helpful to you? (check all that apply)
	• Policy statements provided by my professional associations.
	• Continuing medical education (CME) on climate change and health.
	• Patient education materials.
	• Guidance on how to make the workplace sustainable.
	• Training to communicate effectively about climate and health.
	• Action Alerts - (timely information) on when and how to advocate with policy makers.
	• Podcasts on climate change and health.
	• Other resources.
	• I am not interested in any resources.
18.	Is there anything else you would like us to know? (free text response)
